# Synthesis and evaluation of [^18^F]FBNAF, a STAT3-targeting probe, for PET imaging of tumor microenvironment

**DOI:** 10.1186/s41181-024-00276-w

**Published:** 2024-06-04

**Authors:** Anna Miyazaki, Yasukazu Kanai, Keita Wakamori, Serina Mizuguchi, Mikiya Futatsugi, Fuko Hirano, Naoya Kondo, Takashi Temma

**Affiliations:** 1 Department of Biofunctional Analysis, Graduate School of Pharmaceutical Sciences, Osaka Medical and Pharmaceutical University, 4-20-1 Nasahara, Takatsuki, Osaka 569-1094 Japan; 2 BNCT Joint Clinical Institute, Osaka Medical and Pharmaceutical University, 2-7 Daigaku-Machi, Takatsuki, Osaka 569-8686 Japan

**Keywords:** STAT3, [^18^F]FBNAF, Inhibitor, Imaging probe

## Abstract

**Background:**

Signal transducer and activator of transcription 3 (STAT3) is a protein that regulates cell proliferation and differentiation, and it is attracting attention as a new index for evaluating cancer pathophysiology, as its activation has been highly correlated with the development and growth of tumors. With the development of STAT3 inhibitors, the demand for imaging probes will intensify. Noninvasive STAT3 imaging can help determine the cancer status and predict the efficacy of STAT3 inhibitors. In this study, we aimed to develop an imaging probe targeting STAT3 and synthesized [^18^F]FBNAF, which was derived from a STAT3-selective inhibitor as the lead compound, followed by in vitro and in vivo evaluations of [^18^F]FBNAF in positron emission tomography for STAT3.

**Results:**

The results revealed that FBNAF concentration-dependently inhibited STAT3 phosphorylation, similar to the lead compound, thereby supporting radiosynthesis. [^18^F]FBNAF was easily synthesized from the pinacol boronate ester precursor with suitable radiochemical conversion (46%), radiochemical yield (6.0%), and radiochemical purity (> 97%). [^18^F]FBNAF exhibited high stability in vitro and in vivo, and radioactivity accumulated in tumor tissues expressing STAT3 with an increasing tumor/blood ratio over time, peaking at 2.6 ± 0.8 at 120 min after injection in tumor-bearing mice. Tumor radioactivity was significantly reduced by the coinjection of a STAT3-selective inhibitor. Furthermore, the localization of radioactivity was almost consistent with STAT3 expression based on ex vivo autoradiography and immunohistochemistry using adjacent tumor sections.

**Conclusions:**

Thus, [^18^F]FBNAF could be the first promising STAT3-targeting probe for PET imaging. A STAT3 imaging probe provides meaningful information on STAT3-associated cancer conditions and in tumor microenvironment.

**Supplementary Information:**

The online version contains supplementary material available at 10.1186/s41181-024-00276-w.

## Background

With the development of molecular biology, molecular targeted drugs for various diseases, such as cancer and autoimmune diseases, have been actively developed recently. Identifying critical disease factors has made it possible to develop specific medicines and treat diseases more efficiently with minimal side effects. Cancer treatment has made great advances in recent years, including the development of superior medicines, advanced medical technologies, and radiation therapy. Therefore, cancer will soon be recognized as a curable disease. However, the effectiveness of such treatments remains limited to specific types and stages of cancers; therefore, a definitive diagnosis is required, especially within the early phase of disease, to optimize treatment. If an accurate diagnosis is delayed and metastases or mutations occur before treatment is initiated, the outcome is naturally worse. In this context, nuclear medical techniques such as positron emission tomography (PET) and single-photon emission computed tomography could be optimal diagnostic modalities for this purpose because they can noninvasively image functional molecules inside the human body with high sensitivity and quantitativity, providing detailed information about the pathophysiology during the early phase (Weissleder and Mahmood 2001; Thakur and Lentle 2005).

Signal transducer and activator of transcription 3 (STAT3) is related to cancer development, proliferation, and metastasis. The binding of cytokines such as interleukin (IL)-6 and epidermal growth factor (EGF) to their receptors on the cell membrane leads to the phosphorylation of Janus kinase (JAK), followed by the activation of STAT3 via phosphorylation at Tyr705 (pSTAT3) (Stepkowski et al. 2008; He et al. 2021). pSTAT3 dimerizes, migrates to the nucleus, and then binds to DNA to activate transcription and promote cancer cell growth and metastasis (Sgrignani et al. 2018). STAT3 activation has been observed in hematological malignancies (Arora et al. 2018) and solid tumors (Yu and Jove 2004), head and neck cancer (Sriuranpong et al. 2003), melanoma (Niu et al. 2002), lung cancer (Song et al. 2003), pancreatic cancer (He et al. 2021), breast cancer (Garcia et al. 2001), prostate cancer (Mora et al. 2002; Lou et al. 2000), and ovarian cancer (Liang et al. 2020). STAT3 phosphorylation is also enhanced in the tumor microenvironment, creating an immunosuppressive environment (Zou et al. 2020). Activated STAT3 within the tumor microenvironment induces the malignant transformation of relevant cells to immunosuppressive forms, such as M2-like tumor-associated macrophages, Tregs, and cancer-associated fibroblasts, thereby promoting cancer growth, expansion, and infiltration (Zou et al. 2020; Albrengues et al. 2015; Verdeil et al. 2019). Activated STAT3 inhibits the differentiation and maturation of dendritic cells by activating immunosuppressive factors such as IL-10, vascular endothelial growth factor, and transforming growth factor-β, leading to immune evasion (Yu and Jove 2004). Activated STAT3 then weakens the function of natural killer cells and CD8^+^ T cells, thereby suppressing the antitumor T cell response (Zou et al. 2020). Overall, STAT3 critically plays an essential role in the occurrence and growth of cancers.

**Fig. 1:**
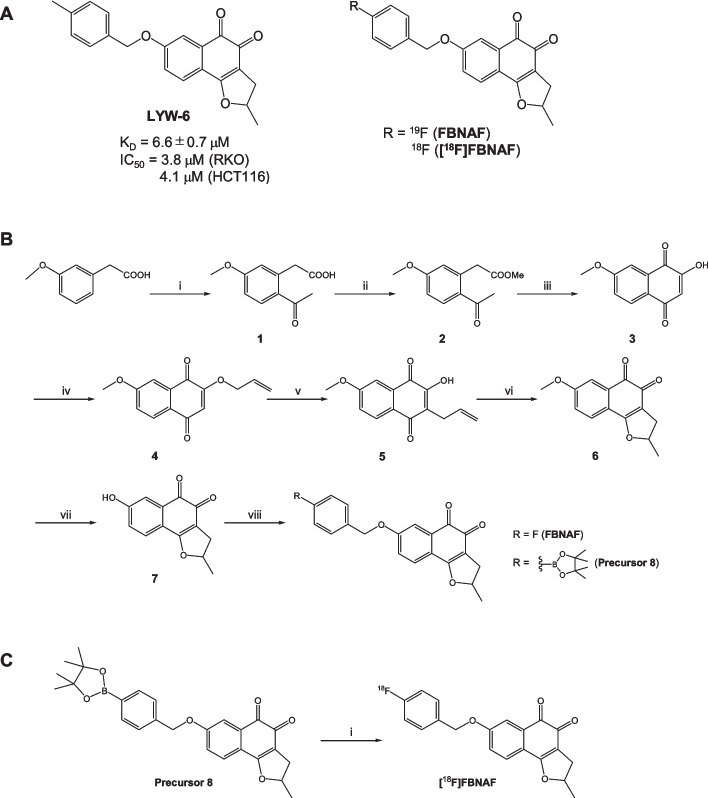
**A** The structures of LYW-6 and the developed compounds FBNAF and [^18^F]FBNAF. **B** Synthesis of FBNAF and the pinacol boronate ester precursor. Reagents and conditions: (i) AlCl_3_, AcCl, dry DCM, 0 °C → room temperature, overnight; (ii) AcCl, dist*.* MeOH, reflux, 4 h; (iii) MeONa, dist. MeOH, reflux, 4 h; (iv) K_2_CO_3_, allyl bromide, DMF, room temperature → 50 °C, 3 h; (v) toluene, reflux, 6 h; (vi) NbCl_5_, dry DCM, 4 h; (vii) AlCl_3_, dry DCM, reflux overnight; (viii) K_2_CO_3_, 4-fluorobenzyl bromide or 4-(4,4,5,5-tetramethyl-a,3,2-dioxaborolan-2-yl)benzyl bromide, dry DMF, 80 °C, 1 h. **C** Radiochemical synthesis. Reagents and conditions: (i) [Cu(OTf)_2_(py)_4_], [^18^F]KF/K_222_, dry DMA, 110 °C, 20 min

STAT3 inhibitors have been developed as promising antitumor drugs. It was indicated that STAT3 inhibitors work well for various cancers occurring in different organs (Zou et al. 2020). Meanwhile, cross-sectional profiling of cancer focusing on the expression and function of STAT3 will provide significant information on the characteristics, immunological status, and metastatic potential of cancers, helping to assess the effectiveness of STAT3 inhibitors for cancer treatment. At present, pan-cancer analysis of STAT3 based on cancer genome databases has attracted attention, and it has been reported that STAT3 can function as a prognostic and immunological biomarker (He et al. 2023). On these bases, we aimed to develop an ^18^F-labeled molecular imaging probe for in vivo PET imaging of STAT3 in cancers because there has been no research on STAT3 imaging probes. Such molecular imaging probes will facilitate evaluation of the physiological roles of STAT3 in vivo, prediction of drug efficacy for personalized medicine, and diagnosis of pathological conditions. We designed [^18^F]FBNAF based on the potent STAT3 inhibitor LYW-6 (Wang et al. 2020), as shown in Fig. 1A. LYW-6 binds to the SH2 domain of STAT3 (K_D_ = 6.6 μM) and inhibits Tyr705 phosphorylation independently of upstream kinases such as JAK. LYW-6 inhibits STAT3 phosphorylation directly and selectively without affecting normal cells. We substituted the methyl group of LYW-6 with a small [^18^F]F atom to minimize the effect on activity. In this study, we synthesized [^18^F]FBNAF and evaluated its properties by phosphorylation inhibitory activity, in vitro stability, in vivo biodistribution, and ex vivo autoradiographic analyses.

## Methods

### General

All reagents were obtained commercially and used without further purification. Low-resolution mass spectrometry (LRMS) was performed using an ACQUITY Ultra Performance LC (Waters Co., Milford, MA, USA) in electrospray ionization mode, and high-resolution mass spectrometry (HRMS) was performed using a JMS-700 mass spectrometer (JEOL, Ltd., Tokyo, Japan) in the electron ionization mode. Nuclear magnetic resonance (NMR) spectra were recorded using a JNM-ECZL400S (JEOL, Ltd.). A thin-layer chromatography (TLC) plate (TLC silica gel 60F_254_, Merck, Darmstadt, Germany) was detected with ultraviolet (UV) light at 254 nm. High-performance liquid chromatography (HPLC) was performed on an LC-20AT (Shimadzu, Kyoto, Japan) equipped with an SPD-20A (Shimadzu) UV detector and a 170 radioisotope detector (Beckman) using COSMOSIL 5C_18_-AR-II column (10 × 150 mm; Nacalai Tesque, Inc., Kyoto, Japan) for reverse-phase chromatography. Precursor **8** for [^18^F]FBNAF and nonradioactive FBNAF were prepared from compound **7**. The structures were confirmed by NMR analysis and HRMS (Supplementary Fig. S1).

### Radiosynthesis

[^18^F]HF was produced using an in-house 20 MeV cyclotron (CYPRIS HM-20, Sumitomo Heavy Industry Co. Ltd., Tokyo, Japan) by the ^18^O(p, n)^18^F nuclear reaction of ^18^O-enriched water. The beam current was 20 μA, and the irradiation time was approximately 5 min. [^18^F]HF was trapped on a Sep-Pak Accell Plus QMA Plus Light Cartridge (WAT023525, Waters) and eluted with K_2_CO_3_/H_2_O (4 mg/200 μL) and K_222_/CH_3_CN (13 mg/800 μL) mixed solution into a vial. The solvent was evaporated at 100℃, followed by drying azeotropically with dry CH_3_CN (1 mL) three times. The residue was dissolved in dry *N,N*-dimethylacetamide (DMA) (400 μL) and transferred to a conical vial containing precursor **8** (3.0 mg, 6.7 μmol) and [Cu(OTf)_2_(py)_4_] (9.1 mg, 13 μmol). The reaction mixture was heated at 110℃ for 20 min. After cooling to quench and filtration through a Cosmonice filter W (pore size: 0.45 μm; 06543–04, Nacalai Tesque), the crude product was purified by reverse-phase HPLC (RP-HPLC) [water: CH_3_CN = 70:30 (initial), 40:60 (5 min), 20:80 (15 min), flow rate = 5 mL/min]. The purified product was evaporated and dissolved in 5% DMSO in PBS ( −) to give [^18^F]FBNAF.

### Cell culture

Human A549 lung cancer cells were obtained from ATCC (Manassas, VA, USA) and cultured in Dulbecco’s Modified Eagle Medium (high glucose) containing 10% fetal bovine serum and 1% penicillin/streptomycin. Cells were incubated in a humidified atmosphere at 37℃ with 5% CO_2_.

### Animals

BALB/c nude mice (male, 4 weeks old) and ddY mice (male, 6 weeks old) were purchased from Japan SLC (Shizuoka, Japan) and given free access to food and water. All animal experiments were conducted according to Osaka Medical and Pharmaceutical University guidelines, and the study protocol was approved by the Institutional Experimental Animal Committee (Permission Number: 22-76, AP23-004). To prepare tumor-bearing mice, A549 cells (4 × 10^6^ cells/mouse) suspended in culture medium (100 μL) were implanted subcutaneously into the right hind leg of each mouse. Animals with a tumor size of approximately 100 mm^3^ (length × width^2^ × 1/2) after inoculation with A549 cells were used for in vivo and ex vivo experiments.

### In vitro stability

[^18^F]FBNAF (152 kBq, 20 μL) in 5% DMSO/PBS ( −) was incubated with ddY mouse plasma (180 μL) at 37℃ for 10, 60, 120, or 180 min. After protein removal using CH_3_CN (400 μL), the supernatant was filtered through a Cosmonice Filter W and analyzed by TLC (Rf = 0.4, ethyl acetate:hexane = 1:1), followed by radioactivity counting using a γ-counter (2480 Wizard^2^, PerkinElmer Japan, Kanagawa, Japan; n = 3). The intact rate (%) was represented as the intact form relative to the total radioactivity.

### Phosphorylation inhibitory assay

Several concentrations of FBNAF (final conc. 0, 0.2, 0.5 1.0, and 2.0 μM in medium containing 1% DMSO) were added to A549 cells, followed by incubation at 37℃ for 24 h. After harvesting, cells were lysed with Passive Lysis Buffer (Promega, Madison, WI, USA) containing 1% protease inhibitor and 1% phosphatase inhibitor. Cell protein was quantified using a Pierce BCA Protein Assay Kit (Thermo Scientific, Waltham, MA, USA). The samples (15 μg/lane) were loaded and separated on an sodium dodecyl sulfate (SDS) polyacrylamide gel. Western blotting was performed using primary antibodies against STAT3 (1:2000 dilution, #4904, Cell Signaling Technology, Danvers, MA, USA), pSTAT3(Tyr705) (1:2000 dilution, #9145, Cell Signaling Technology), and β-actin (1:5000 dilution, NB600-503, Novus Biologicals, Centennial, CO, USA), as well as anti-rabbit IgG HRP-conjugated antibody (1:3000 dilution, HAF008, R&D Systems, Minneapolis, MN, USA) as a secondary antibody. The western blot bands were detected using Chemi-Lumi One L (Nacalai Tesque) and analyzed using an Amersham imager 600 (Cytiva, Tokyo, Japan). To detect pSTAT3, Chemi-Lumi One Ultra (Nacalai Tesque) was used.

### In vivo biodistribution and blocking studies

[^18^F]FBNAF (115-343 kBq, 100 μL) in 5% DMSO/PBS (−) was intravenously injected into mice. The animals were euthanized under isoflurane anesthesia at 10, 30, 60, and 120 min postinjection, and the tissues of interest were excised and weighed. Radioactivity was measured using a γ-counter. The accumulation of radioactivity was expressed as the percent injected dose per gram tissue (%ID/g) for all tissues excluding the stomach, in which accumulation was expressed as the percent injected dose per organ (%ID). For the blocking study, [^18^F]FBNAF (115-360 kBq, 100 μL) in 5% DMSO/PBS (−) was intravenously coinjected with BP-1-102 (3.0 mg/kg, Selleck, Shanghai, China) into mice, and after 30 min, the accumulated radioactivity in the tumor, blood, and muscle were measured. Radioactivity was decay-corrected at the time of administration.

### Ex vivo autoradiography and immunohistochemistry

Tumors were excised 30 min after the intravenous injection of [^18^F]FBNAF (3–6 MBq, 100 μL) and immediately frozen in OCT compound. The frozen tumor was cut into 10 µm thick sections using Cryotome and contacted with a phosphor screen in a cassette overnight. The screen was scanned using an Amersham Typhoon scanner (Cytiva), and the resulting images were analyzed using Image-Quant TL (Cytiva).

For immunohistochemistry, adjacent sections were fixed with cold acetone (− 20℃) for 10 min. After blocking using Blocking One Histo (Nacalai Tesque), the sections were incubated with antibodies against STAT3 (1:200 dilution, #4904) or pSTAT3(Tyr705) (1:200 dilution, #9145) overnight at 4℃. After a rinse, anti-rabbit IgG(H + L) CF555 (1:1000 dilution, SAB4600068, Sigma-Aldrich, St. Louis, MO, USA) was added, followed by incubation for 30 min at room temperature. Nuclear staining was performed by incubation with Hoechst 33,342 (Nacalai Tesque) for 10 min. The sections were observed with a BZ-X810 fluorescence microscope (Keyence Japan Co., Osaka, Japan) and analyzed using a BZ-X800 Analyzer (Keyence Japan Co.)

### Statistical analysis

All experiments were performed with a sample size of n ≥ 3, and data are expressed as the mean ± standard deviation (SD). Multiple comparisons of the phosphorylation inhibitory experiment were analyzed by the Kruskal–Wallis one-way ANOVA and Dunn’s post hoc test using GraphPad Prism 8 (GraphPad Software Inc., La Jolla, CA, USA). For the blocking study, the unpaired Mann–Whitney *U* test was used. One-tailed *p* < 0.05 was considered statistically significant.

## Results

### Synthesis

Intermediates **1–7** were prepared as described by Wang et al*.* (2020), Caron et al. (2007), Bian et al. (2015) and Khmelevskaya and Pelageev (2019). Nonradioactive FBNAF was successfully obtained by Williamson ether synthesis (Fig. 1B). The chemical purity of FBNAF exceeded 95% after purification by RP-HPLC. In addition, the pinacol boronate ester precursor **8** was prepared from compound **7** and 4-(4,4,5,5-tetramethyl-a,3,2-dioxaborolan-2-yl)benzyl bromide for the radiosynthesis of [^18^F]FBNAF. All compounds were characterized using ^1^H NMR, LRMS, and HRMS. These detailed synthetic methods and data are described in Supplementary Fig. S1.

### Phosphorylation inhibition in vitro

**Fig. 2:**
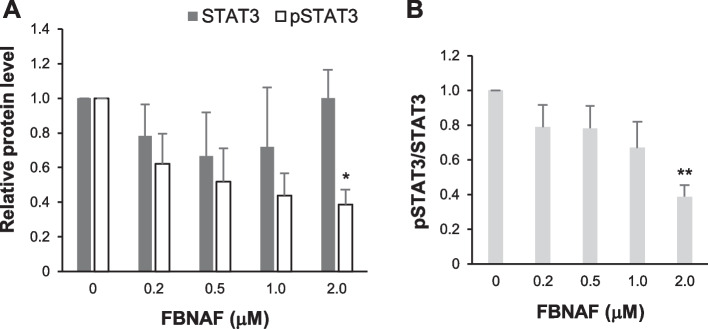
FBNAF inhibited STAT3 phosphorylation (Tyr705). Western blotting of STAT3, pSTAT3 (Tyr705), and β-actin in A549 cells treated with 0–2 μM FBNAF for 24 h. **A** STAT3 and pSTAT3 expression was quantified and represented as relative protein levels against β-actin as a loading control. The respective relative protein level in the untreated cells was given as 1. **B** the ratio of pSTAT3 to STAT3 was calculated from A. Representative full-length gel and western blot bands are presented in the supporting information (Fig. S2). The error bars denote the SD (n = 3). *p < 0.05, **p < 0.01 versus control (0 μM FBNAF) calculated by the Kruskal–Wallis test

To examine whether FBNAF inhibits STAT3 phosphorylation (Tyr705), we performed western blotting using human A549 lung cancer cells, which highly express STAT3 (Song et al. 2003). Total STAT3 and pSTAT3 levels in cells after treatment with the compound (0–2 μM) were quantitatively analyzed (Fig. 2, Supplementary Fig. S2). FBNAF inhibited pSTAT3 expression in a concentration-dependent manner, including 50% and 60% inhibition at concentrations of 0.5 and 1.0 μM, respectively (Fig. 2A). At the same time, no significant change was observed in total STAT3 expression. Thus, the ratio of pSTAT3 to total STAT3 decreased as the concentration of FBNAF increased (Fig. 2B). This result is similar to that of LYW-6, as reported by Wang et al. (2020), suggesting that the minor structural modification to LYW-6 did not diminish its phosphorylation inhibitory activity.

### Radiosynthesis

**Fig. 3:**
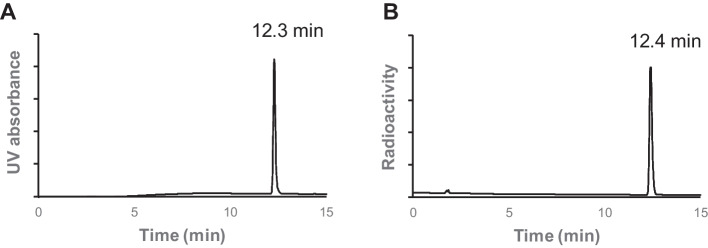
HPLC analysis of FBNAF (**A**) and [^18^F]FBNAF (**B**). HPLC condition: COSMOSIL 5C18 AR-II column (10 mm I.D. × 250 mm) using water/CH_3_CN [v/v = 70/30 (initial), 40/60 (5 min), 20/80 (15 min)], flow 5 mL/min

[^18^F]FBNAF was synthesized in one step from the precursor **8** via manual radiosynthesis (Fig. 1C). The ester was radiofluorinated under a no-carrier-added condition in the presence of [^18^F]KF/Kryptofix_222_ (K_222_), K_2_CO_3_, and [Cu(OTf)_2_(py)_4_] to give [^18^F]FBNAF at a radiochemical conversion (RCC) of 46 ± 17% (n = 6, from precursor **8** to [^18^F]FBNAF). The overall decay-corrected radiochemical yield (RCY) (n = 6, from [^18^F]HF to the end of synthesis) was 6.0 ± 3.3% after purification by RP-HPLC and formulation for animal experiments. The total operation time was within 150 min. The purified [^18^F]FBNAF was isolated by RP-HPLC and identified by additional RP-HPLC analysis with a coincidence of retention times between the UV absorbance of FBNAF and radioactivity of [^18^F]FBNAF (Fig. 3). The radiochemical purity was greater than 97%.

### In vitro stability analysis

**Fig. 4:**
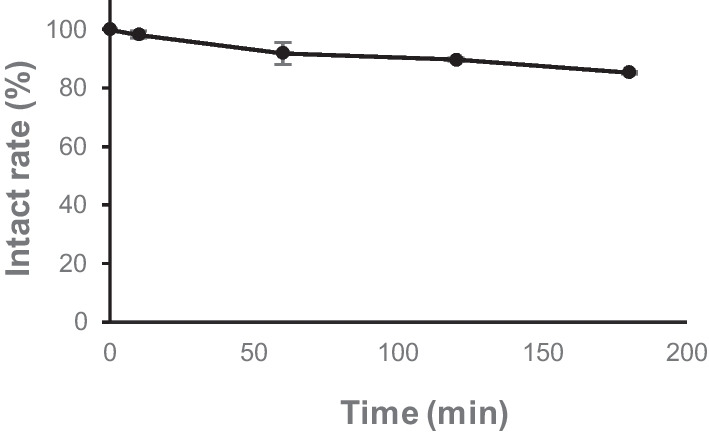
Stability of [^18^F]FBNAF in mouse plasma. The intact rate (%) of [^18^F]FBNAF was examined by normal-phase radio-TLC after incubating [^18^F]FBNAF (20 μL, 152 kBq) in ddY mouse plasma (180 μL, n = 3)

As shown in Fig. 4, more than 85% of the radioactivity was detected in an intact form by normal-phase TLC analysis after 3 h of incubation in mouse plasma. The high stability of [^18^F]FBNAF was confirmed in vitro, supporting the validity of the tracer for in vivo applications.

### In vivo biodistribution study

We studied the biodistribution of [^18^F]FBNAF in A549 tumor-bearing mice (Table 1). After the intravenous injection of [^18^F]FBNAF, radioactivity was first detected in the liver, kidneys, and small intestine 10 min postinjection, moved to the small intestine at 60 min, and cleared to the colon at 120 min and later, indicating excretion of the radiotracer via the hepatobiliary pathway. This distribution pattern was similar to that in normal mice (Supplementary Table S1). Aside from excretory organs, radioactivity peaked at 10 min in almost all organs and quickly disappeared. Moderate radioactivity was observed in bone over time. The tumor radioactivity peaked at 30 min (1.7 ± 0.4%ID/g), after which it declined at 60 min but maintained to some extent until 120 min later (0.9 ± 0.3%ID/g). Radioactivity was rapidly cleared from blood and muscle, and the radioactivity in blood and muscle finally decreased to 0.3 ± 0.0 and 0.5 ± 0.1%ID/g, respectively, at 120 min. Thus, the tumor-to-blood ratio, which is recognized as an imaging index, increased over time to reach 2.6 ± 0.8 at 120 min postinjection.

**Table 1:** Biodistribution of [^18^F]FBNAF in A549 tumor-bearing mice

<Tissue > (%ID/g)	Time after injection (min)											
	10			30			60			120		
Blood	1.0 ± 0.2			1.0 ± 0.3			0.5 ± 0.1			0.3 ± 0.0		
Heart	2.3 ± 0.8			1.5 ± 0.7			0.7 ± 0.1			0.7 ± 0.2		
Lung	4.4 ± 1.0			3.3 ± 2.0			1.2 ± 0.0			1.0 ± 0.3		
Liver	10.8 ± 2.8			5.3 ± 1.9			2.2 ± 0.3			1.6 ± 0.8		
Kidney	19.5 ± 7.7			4.5 ± 1.2			0.9 ± 0.3			0.5 ± 0.2		
Stomach*	0.6 ± 0.3			3.6 ± 2.8			1.4 ± 1.4			0.8 ± 1.0		
Small intestine	11.6 ± 5.5			38.3 ± 5.7			48.4 ± 7.4			17.8 ± 2.2		
Colon	0.8 ± 0.4			0.7 ± 0.4			11.0 ± 3.5			44.4 ± 16.3		
Pancreas	6.3 ± 2.9			1.1 ± 0.3			0.5 ± 0.1			0.6 ± 0.1		
Spleen	2.7 ± 0.7			1.6 ± 0.5			0.9 ± 0.1			0.8 ± 0.3		
Muscle	2.1 ± 0.8			0.6 ± 0.1			0.5 ± 0.2			0.5 ± 0.1		
Bone	2.1 ± 0.2			2.3 ± 0.6			1.5 ± 0.5			1.9 ± 0.2		
Brain	1.9 ± 1.1			0.8 ± 0.1			0.5 ± 0.0			0.4 ± 0.1		
Tumor	1.5 ± 0.3			1.7 ± 0.4			0.9 ± 0.3			0.9 ± 0.3		
< Ratios >												
Tumor/blood	1.6 ± 0.3			2.0 ± 1.0			2.0 ± 0.8			2.6 ± 0.8		
Tumor/muscle	0.8 ± 0.1			2.6 ± 0.3			2.4 ± 1.2			1.9 ± 0.9		

Values were represented as % injected dose per gram (%ID/g). The data were expressed as the means ± SD (n = 3 or 4). *Values *were* *represented* *as* *%ID*

### In vivo blocking study

**Fig. 5:**
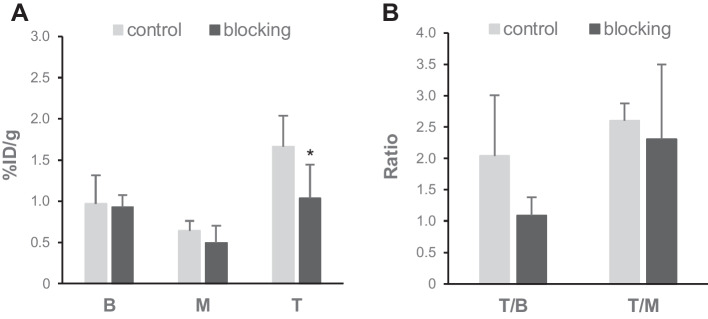
Blocking effect of coinjected BP-1–102.** A** Radioactivity accumulation in the blood (B), muscle (M), and tumor (T) at 30 min after the injection of [^18^F]FBNAF with (blocking group) or without (control group) BP-1–102. **B** Tumor-to-blood (T/B) and tumor-to-muscle (T/M) accumulation ratios at 30 min after the injection of [^18^F]FBNAF. The error bars denote the SD, n = 5. *p < 0.05 versus the control group calculated by the Mann–Whitney *U* test

Next, we evaluated the in vivo radioactivity distribution of [^18^F]FBNAF with or without coinjection of BP-1–102, an orally bioavailable and selective STAT3 inhibitor that binds to the SH2 domain of STAT3 (Zhang et al. 2012), as summarized in Fig. 5A. At 30 min after administration, the tumor radioactivity in the blocking group was significantly lower than that in the control group (1.0 ± 0.3% ID/g vs. 1.5 ± 0.1% ID/g, *p* < 0.05), whereas that in blood and muscle was negligibly affected by coinjection of the inhibitor. The tumor-to-blood ratio was considerably lower in the blocking group than the control group, but the difference was not significant (1.1 ± 0.3 vs. 2.0 ± 1.0, *p* = 0.08, Fig. 5B). This might be attributable to the limited numbers of tested animals (n = 4–5). A slight and nonsignificant decrease was observed in the tumor-to-muscle ratio in the blocking group (2.3 ± 1.2 vs. 2.6 ± 0.3).

### Ex vivo autoradiography and immunohistochemistry

**Fig. 6:**
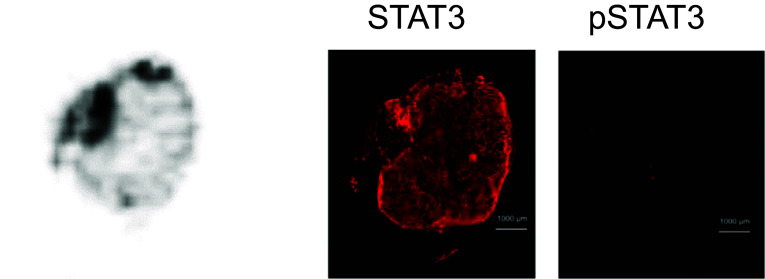
Ex vivo autoradiography and immunohistochemical staining in a tumor section removed from a A549 tumor-bearing mouse administered [^18^F]FBNAF. An autoradiogram was obtained from the tumor excised at 30 min after the injection of [^18^F]FBNAF. Adjacent sections were used for immunohistochemistry with antibodies against STAT3 and pSTAT3. The results of Hoechst staining are also provided in the supporting information (Fig. S3). The scale bar represents 1000 μm

To further ensure that [^18^F]FBNAF could bind to STAT3 in vivo, we performed ex vivo autoradiography using sections prepared from the excised tumors of A549 tumor-bearing mice at 30 min postinjection of [^18^F]FBNAF and compared the results with that of immunohistochemical analysis of an adjacent section (Fig. 6, Supplementary Fig. S3). The autoradiogram revealed an uneven distribution of radioactivity in the tumor sections. This intratumoral radioactivity distribution almost corresponded to the STAT3 expression profile, suggesting a close relationship between [^18^F]FBNAF accumulation and STAT3 expression in tumors.

## Discussion

In this study, we developed [^18^F]FBNAF as a STAT3 imaging probe for PET based on the direct STAT3 inhibitor LYW-6. STAT3 is overexpressed in some cancerous tissues compared to its expression in normal tissues, and activated STAT3 promotes cancer growth and invasion. The level of STAT3 varies by cell type, and it is related to the clinical stage. STAT3 is valuable as a prognostic biomarker because it is correlated with cancer prognosis, drug resistance, and immune mechanisms (He et al. 2023). Thus, STAT3 imaging probes can visualize cancer pathology and provide essential indicators of tumor progression.

STAT3 is a promising target for both diagnosis and treatment; therefore, many approaches have been used in the development of inhibitors, including inhibitors of extracellular signaling molecules such as EGF and IL-6, inhibitors of intracellular kinases such as JAK and Src, both of which exist upstream of the STAT3 signal cascade, and inhibitors that directly bind to STAT3 (Shih 2020; Furtek et al. 2016). Most direct inhibitors target the SH2 domain, which is responsible for the phosphorylation of STAT3 and the formation of homo- or heterodimers (Hua et al. 2022). Such inhibitors have a tricyclic or equivalent rigid frame as a common pharmacophore, exhibiting a similar binding mode involving interactions with two or three hot spots (Zhang et al. 2012; Shih 2020; Furtek et al. 2016; Hua et al. 2022; Dhanik et al. 2012). It has been reported that the tricyclic structure of LYW-6 also interacts with the phosphorylated Tyr705 site in the SH2 domain, which is constituted by hydrophilic amino acids, including Lys591, Ser611, Ser613, and Arg609. And, the benzyloxy moiety of LYW-6 flexibly interacts with the nearby side pocket to enhance its binding affinity for the protein. Because LYW-6 directly interacts with STAT3 with high affinity, high antitumor activity, and high selectivity among STAT members, we adopted LYW-6 as a lead compound to develop a radiolabeled probe for in vivo STAT3 imaging with PET. We then focused on the benzyloxy moiety rather than the tricyclic component for radiolabeling with a small [^18^F]F atom with the expectation that such a minor chemical modification would have negligible effects on the intermolecular interaction and designed [^18^F]FBNAF. In fact, FBNAF inhibited STAT3 phosphorylation with similar potency as LYW-6 (Wang et al. 2020), supporting the validity of our drug design strategy. The results suggested that the introduction of a fluorine atom into the benzyloxy moiety did not interfere with the electronic and steric binding ability of the compound.

In this study, [^18^F]FBNAF was prepared following a one-step radiosynthesis protocol with [^18^F]HF provided by a cyclotron facility at our institute. Radiofluorination of precursor **8** was successful with a comparable conversion yield (RCC = 46 ± 17%) as described in a previous report using various aryl boronic ester precursors (Tredwell et al. 2014). The RCY (6.0 ± 3.3%) was relatively low because of the inefficient recovery of [^18^F]F^−^ and absorption of [^18^F]FBNAF onto the vial and columns throughout the process. As our aim in this study was to develop a STAT3 imaging probe and evaluate its efficacy, optimization of the labelling was not a priority. Therefore, there is space in radiosynthesis to optimize radioactivity recovery and purification procedures. However, the obtained radioactivity was sufficient for later evaluations. Overall, as the first report to develop a novel radiotracer, smooth and successful radiosynthesis was achieved.

In the biodistribution experiment of [^18^F]FBNAF, radioactivity was rapidly delivered throughout the body and cleared via the hepatobiliary excretion to the intestine, as expected from the high hydrophobicity of the radiotracer estimated from HPLC analysis and the ClogP 2.89 (calculated by ChemDraw Ultra 7.0). Radioactivity peaked later in the tumor than in blood and muscle, which are representative nontarget tissues, suggesting active targeting of [^18^F]FBNAF to the tumor depending on the binding interaction with STAT3 in the xenograft mouse model used in this study. In fact, the tumor-to-blood ratio increased over time, reaching 2.6 at 120 min after injection. The tumor-to-muscle ratio exceeded 2.0 at 30 min after injection because the muscle radioactivity decreased to the background level in the early phase. Such rapid and favorable radioactivity pharmacokinetics and the absence of unexpected accumulation in any part of the body support the potential of [^18^F]FBNAF as an in vivo PET imaging probe with the fluorine-18 having a short half-life of 109.8 min.

We examined whether the accumulation of radioactivity in the tumor was attributable to the interaction of [^18^F]FBNAF with STAT3 using a blocking study with the STAT3-selective inhibitor BP-1–102. BP-1–102 is a dimerization inhibitor of STAT3, and its binding mode to STAT3 is similar to that of LYW-6. BP-1–102 interacts with the binding pocket of the SH2 domain, including the phosphorylated Tyr705 binding region, and also interacts with a third sub-pocket, making its binding capacity stronger than that of LYW-6 (K_D_ values for LYW-6 and BP-1–102 are 6.6 μM and 504 nM, respectively) (Wang et al. 2020; Zhang et al. 2012). BP-1–102 was reportedly administered intravenously and perorally into rodents as an aqueous solution and inhibited tumor growth after repeated intravenous administration in A549 tumor-bearing mice (Zhang et al. 2012). On this basis, we determined the dose of 3 mg/kg in the blocking study that could act on STAT3 sufficiently in vivo. The inhibitor significantly reduced tumor radioactivity accumulation and the tumor-to-blood ratio in the xenografted mice after [^18^F]FBNAF administration, suggesting the specific binding of [^18^F]FBNAF to STAT3 in tumor tissues. No significant difference was found in the tumor-to-blood ratio due to the limited numbers of tested animals (n = 4 or 5). Ex vivo autoradiography and immunohistochemistry revealed that the intratumoral radioactivity distribution tended to correspond to STAT3 expression, further supporting the ability of [^18^F]FBNAF to bind STAT3 in vivo.

Concerning the limitations of this study, we should mention concerns about the expression of STAT3 and pSTAT3 in cancerous tissues. We subcutaneously implanted mice with A549 cells because the cell line constantly expresses STAT3 in vitro and in vivo without any treatment, which is convenient for a fundamental study of our novel radiotracer. However, in the prepared tumor sections in this study, almost no pSTAT3 was observed by immunohistochemical analysis. The literature suggests that the relevant cells inducibly express STAT3 and pSTAT3, corresponding to the pathological situation. Therefore, [^18^F]FBNAF should be further validated in varied model animals exhibiting different STAT3 expression patterns in the future. Concern could also be raised about the in vivo stability of [^18^F]FBNAF owing to some accumulation of radioactivity in bone, where [^18^F]F^−^ physiologically accumulates, whereas high stability is exhibited in vitro. However, bone radioactivity was not high over the experimental period relative to the literature describing in vivo defluorination (Craig et al. 2023; Lütje et al. 2019). This might not indicate the defluorination of [^18^F]FBNAF; instead, it reflects STAT3 expression in bone tissue and bone-derived cells involved in bone formation (Jo et al. 2018). Nevertheless, further research is required to clarify the effects of STAT3 expression on radioactivity accumulation in bone. Lastly, the tumor-to-blood ratio obtained in the biodistribution study could attract attention because it was not sufficient to enable apparent in vivo PET imaging, which generally requires a ratio of at least 3. However, radioactivity accumulation depends on the expression of STAT3 in a targeted site, which was not optimized in the animal model used in this study, as mentioned above. Autologous transplant or patient-derived xenograft animals are considered superior as cancerous disease models reflecting human disease conditions, and these models might exhibit higher STAT3 expression than a simple subcutaneous transplant model. Thus, [^18^F]FBNAF requires further evaluation in such models.

## Conclusion

In conclusion, we designed, synthesized, and evaluated [^18^F]FBNAF as a STAT3-selective imaging probe for PET. To the best of our knowledge, this is the first report on the development of a molecular imaging probe for in vivo PET imaging of STAT3. We synthesized [^18^F]FBNAF with a moderate radiochemical conversion and yield and high radiochemical purity by a one-step radiolabeling procedure. The results revealed the comparable binding affinity of [^18^F]FBNAF as the lead compound, high stability, and favorable pharmacokinetics, including tumor accumulation corresponding to STAT3 expression. Further studies are necessary regarding several aspects, including the optimization of radiosynthesis, in vivo utility in varied model animals, and in vivo PET imaging. Our first success would be precious, and further optimized design would lead to superior imaging probes with apparent efficacy.

Because STAT3 is relevant in both cancer and cancer-related cells, noninvasive STAT3 imaging techniques will contribute to elucidating the spatiotemporal function of STAT3 within the tumor microenvironment and development of STAT3-based personalized medicine.

### Supplementary Information

Supplementary file 1

## Data Availability

All data generated or analyzed during this study are included in this published article and its supplementary information files.

## Abbreviations

DCM: Dichloromethane
DMF: *N,N*-Dimethylformamide
DMA: *N,N*-Dimethylacetamide
EGF: Epidermal growth factor
FBNAF: 7-(4-Fluoro-benzyloxy)-2-methyl-2,3-dihydro-naphtho[1,2-b]furan-4,5-dione
[^18^F]FBNAF: 7-(4-[^18^F]Fluoro-benzyloxy)-2-methyl-2,3-dihydro-naphtho[1,2-b]furan-4,5-dione
HRMS: High-resolution mass spectrometry
HPLC: High-performance liquid chromatography
IL: Interleukin
JAK: Janus kinase
LRMS: Low-resolution mass spectrometry
NMR: Nuclear magnetic resonance
PET: Positron emission tomography
pSTAT3: STAT3 via phosphorylation at Tyr705
RCC: Radiochemical conversion
RCY: Radiochemical yield
RP-HPLC: Reverse-phase HPLC
SDS: Sodium dodecyl sulfate
STAT3: Signal transducer and activator of transcription 3
TLC: Thin-layer chromatography
UV: Ultraviolet
