# Supplementary material for: Synthesis and evaluation of [18F]FBNAF, a STAT3-targeting probe, for PET imaging of tumor microenvironment
Source: EJNMMI Radiopharm Chem. 2024 Jun 4;9:46. doi: 10.1186/s41181-024-00276-w (PMC11150212; doi:10.1186/s41181-024-00276-w)
Supplement: Supplementary file 1 [file 41181_2024_276_MOESM1_ESM.docx]

**Supplementary information**

**Synthesis and evaluation of [^18^F]FBNAF, a STAT3-targeting probe,
for PET imaging of tumor** **microenvironment**

**Anna Miyazaki^1^, Yasukazu Kanai^1, 2^, Keita Wakamori^1^, Serina Mizuguchi^1^, Mikiya Futatsugi^1^, Fuko Hirano^1^, Naoya Kondo^1^, Takashi Temma^1,*^**

^1^Department of Biofunctional Analysis, Graduate School of Pharmaceutical Sciences, Osaka Medical and Pharmaceutical University, 4-20-1 Nasahara, Takatsuki, Osaka 569-1094, Japan.

^2^BNCT Joint Clinical Institute, Osaka Medical and Pharmaceutical University, 2-7, Daigaku-Machi, Takatsuki, Osaka 569-8686, Japan.

*Correspondence: Takashi Temma ([takashi.temma@ompu.ac.jp](mailto:takashi.temma@ompu.ac.jp))

Contents:

Synthetic method and data S2

**Fig. S1.** NMR data S3

**Fig. S2.** Phosphorylation inhibitory activity S4

**Fig. S3.** Immunohistochemistry S5

**Table S1.** Distribution study in normal mice S6

**Synthetic method and data**

To a solution of a compound **7** (0.08 mmol) in dry DMF (1 mL) was added 4-fluorobenzyl bromide (0.09 mmol) or 4-(4,4,5,5-tetramethyl-a,3,2-dioxaborolan-2-yl)benzyl bromide (0.09 mmol), and K_2_CO_3_ (0.16 mmol) at room temperature. The solution was stirred at 80℃ for 1 h. The reacted mixture was extracted with ethyl acetate, and the organic layer collected was dried using Na_2_SO_4_ and concentrated. The given compounds were purified like below.

***7-(4-Fluoro-benzyloxy)-2-methyl-2,3-dihydro-naphtho[1,2-b]furan-4,5-dione (FBNAF).***

The crude was purified by RP-HPLC to give FBNAF as a red powder (> 99% purity). Yield 46%, ^1^H NMR (400 MHz, DMSO-*d*_6_) δ: 7.50-7.60 (m, 4H), 7.36 (dd, 1H, *J* = 2.8 and 9.8 Hz), 7.24 (t-like, 2H, *J* = 8.6 Hz), 5.30 (m, 1H), 5.25 (s, 2H), 3.13 (dd, 1H, *J* = 9.6 and 14.8 Hz), 2.57 (dd, 1H, *J* = 6.8 and 15 Hz), 1.49 (d, 3H, J = 6.4 Hz). HRMS-EI (m/z): calcd for C_20_H_15_FO_4_ (M+) 338.0954, found for 338.0953.

***2-Methyl-7-[4-(4,4,5,5-tetramethyl-[1,3,2]dioxaborolan-2-yl)-benzyloxy]-2,3-dihydro-naphtho[1,2-b]furan-4,5-dione (8).***

The crude was purified by flash column chromatography to give compound **8** as red powder. Yield 42%, ^1^H NMR (400 MHz, DMSO-*d*_6_) δ: 7.69 (d, 2H, *J* = 8.0 Hz), 7.56 (d, 1H, *J* = 8.4 Hz), 7.48 (d, 1H, *J* = 2.8 Hz), 7.46 (d, 2H, *J* = 8.0 Hz), 7.33 (dd, 1H, *J* = 2.4 and 8.4 Hz), 5,30 (s, 2H), 5.27 (m, 1H), 3.11 (dd, 1H, *J* = 9.6 and 14.8 Hz), 2.55 (dd, 1H, *J* = 6.8 and 14.8 Hz), 1.47 (d, 3H, *J* = 6.0 Hz), 1.28 (s, 12H). HRMS-EI (m/z): calcd for C_26_H_27_BO_6_ (M+) 446.1901, found for 446.1906.

**
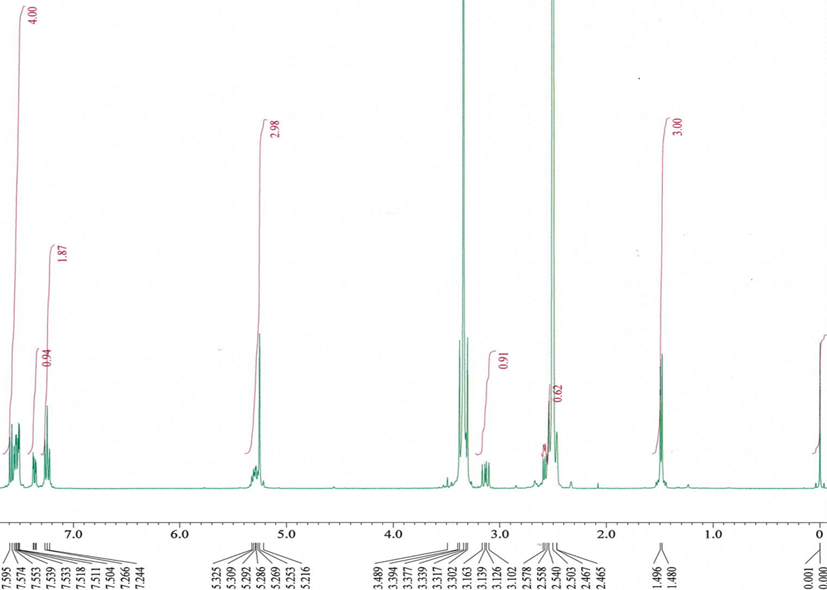
A**


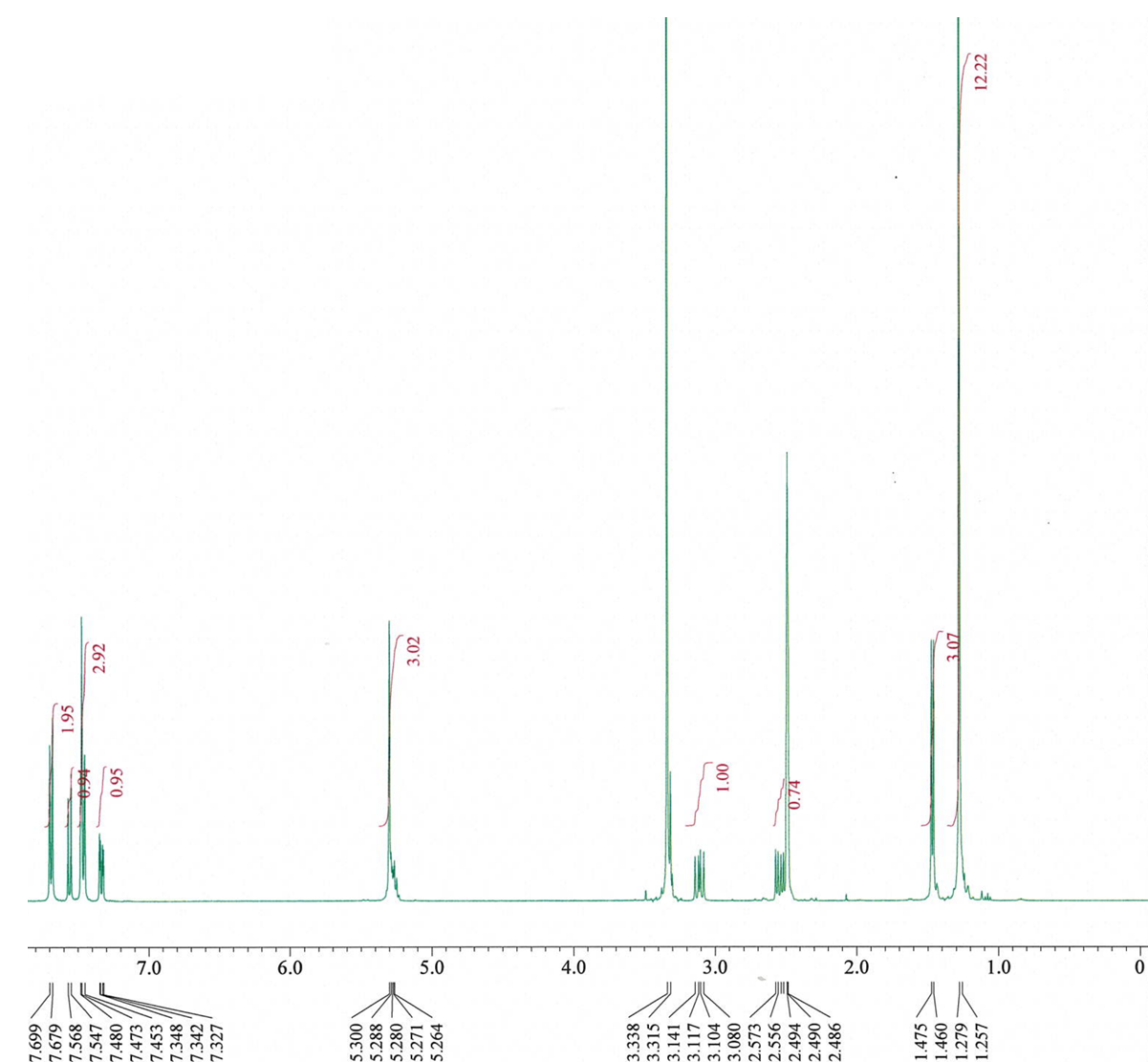
**B**

**Fig. S1.** ^1^H-NMR data (400 MHz, DMSO-*d*_6_). **A** FBNAF; **B** the precursor 8


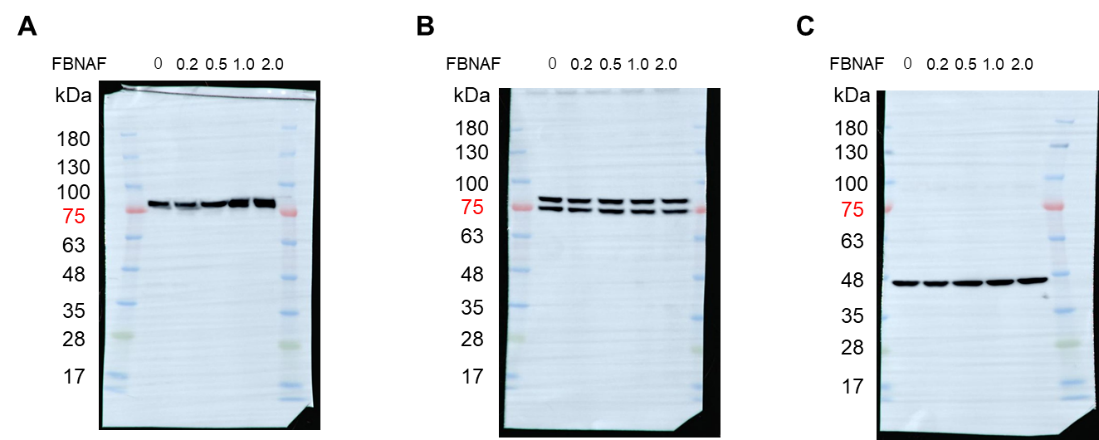


**Fig. S2.** Representative western blot analysis in full-length gels. **A** STAT3 (86 kDa); **B** pSTAT3 (Tyr705) isoforms, α (86 kDa) and β (79 kDa); **C** β-actin (42 kDa) in A549 cells treated with 0-2 μM of FBNAF for 24h

**
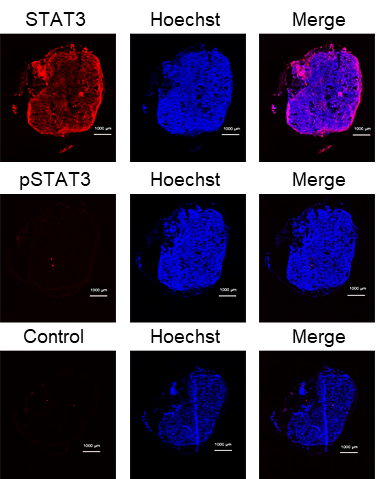
**

**Fig. S3.** Immunohistochemical staining in a tumor section removed from A549 bearing mouse. Tumor sections were stained with an antibody for STAT3, pSTAT3, and Hoechst, respectively. The scale bar represents 1000 μm

**
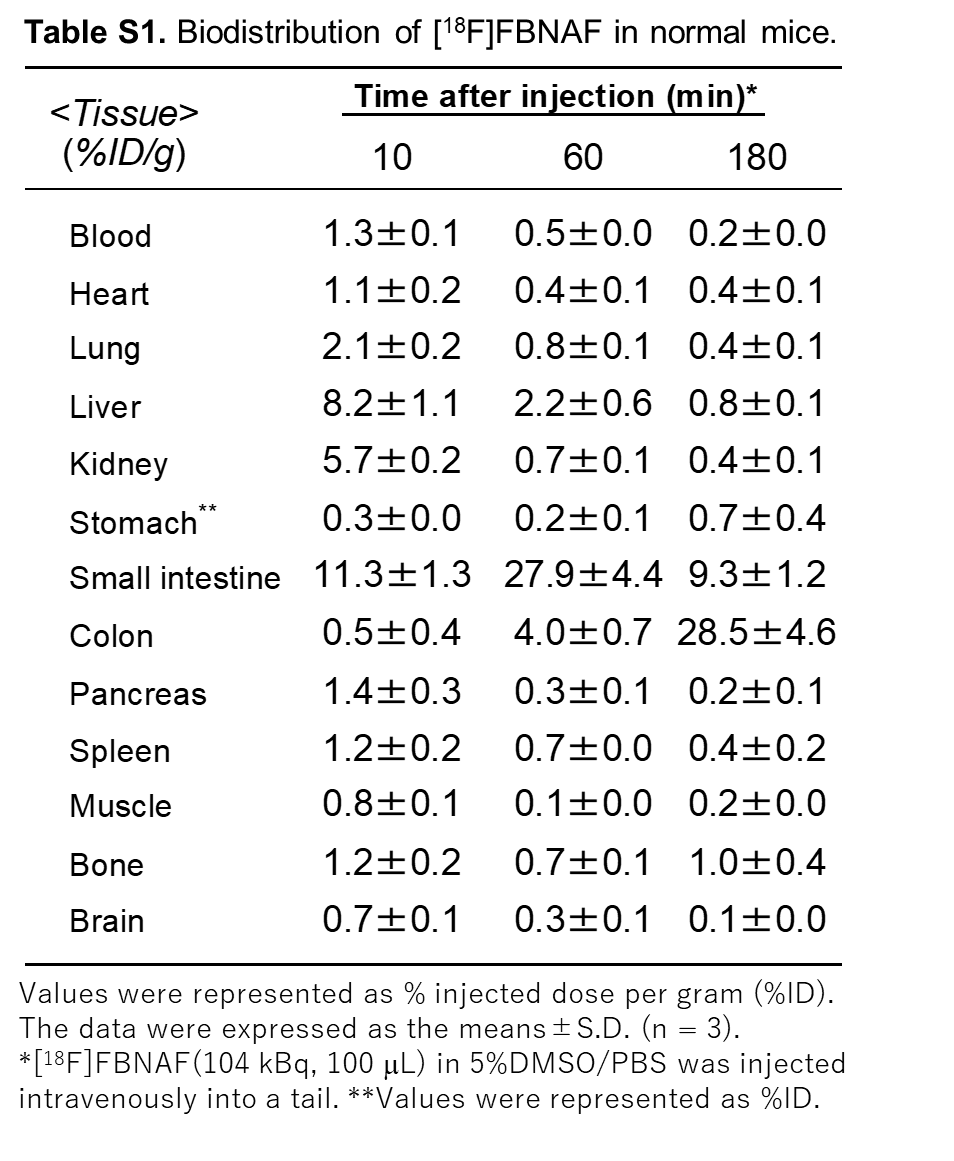
**
